# Association of Nonalcoholic Fatty Liver Disease and Fibrosis With Incident Dementia and Cognition: The Rotterdam Study

**DOI:** 10.1212/WNL.0000000000200770

**Published:** 2022-08-09

**Authors:** Tian Xiao, Laurens van Kleef, M. Kamran Ikram, Robert De Knegt, M. Arfan Ikram

**Affiliations:** From the Departments of Epidemiology (T.X., M.A.I.), Gastroenterology and Hepatology (L.K., R.D.K.), Neurology (M.K.I.), Erasmus MC University Medical Center, Rotterdam, the Netherlands.

## Abstract

**Background and Objectives:**

Nonalcoholic fatty liver disease (NAFLD) might affect brain health via the so-called liver-brain axis. Whether this results in an increased risk for dementia remains unclear. Therefore, we investigated the association of NAFLD and fibrosis with incident dementia and cognition among the elderly.

**Methods:**

We performed longitudinal and cross-sectional analyses within the Rotterdam Study, an ongoing prospective cohort. Participants visiting between 1997 and 2002 with available fatty liver index (FLI) (set 1) or participants visiting between 2009 and 2014 with abdominal ultrasound (set 2) and liver stiffness (set 3) were included. Exclusion criteria were secondary causes for steatosis, prevalent dementia, and missing alcohol data. NAFLD was defined as FLI ≥60 or steatosis on ultrasound and fibrosis as liver stiffness ≥8.0 kPa. Dementia was defined according to the *DSM-III-R*. Associations between NAFLD, fibrosis, or liver stiffness and incident dementia were quantified using Cox regression. Finally, the association between NAFLD and cognitive function was assessed cross-sectionally.

**Results:**

Set 1 included 3,975 participants (age 70 years, follow-up 15.5 years), set 2 4,577 participants (age 69.9 years, follow-up 5.7 years), and set 3 3,300 participants (age 67.6 years, follow-up 5.6 years). NAFLD and fibrosis were consistently not associated with an increased risk for dementia (NAFLD based on ultrasound, hazard rate [HR] 0.84, 95% CI 0.61–1.16; NAFLD based on FLI, HR 0.92, 95% CI 0.69–1.22; fibrosis, HR 1.07, 95% CI 0.58–1.99) in fully adjusted models. Of interest, NAFLD was associated with a significantly decreased risk for incident dementia until 5 years after FLI assessment (HR 0.48; 95% CI 0.24–0.94). Moreover, NAFLD was not associated with worse cognitive function, covering several domains.

**Conclusions:**

NAFLD and fibrosis were not associated with an increased risk for incident dementia, nor was NAFLD associated with impaired cognitive function. In contrast, NAFLD was even protective in the first 5 years of follow-up, hinting toward NAFLD regression before dementia onset.

**Trial Registration Information:**

Clinical Trial Number: NTR6831.

Nonalcoholic fatty liver disease (NAFLD) is increasingly common and affects >25% of the global population.^1^ It has become one of the most prevalent chronic liver diseases, ranging from simple fat accumulation to liver cirrhosis.^2^ In addition, recent studies indicate that NAFLD is associated with kidney dysfunction.^3,4^ cardiovascular disease,^5^ and extrahepatic malignancies such as colon and stomach cancer.^6,7^ However, its link with neurodegenerative conditions, such as dementia or cognition impairment, remains unclear.

As a metabolic disease, NAFLD has several risk factors in common with dementia, for example, insulin resistance, hypertension, obesity, physical inactivity, and dyslipidemia.^8^ Accumulating evidence also suggests a direct association of NAFLD with brain structural changes via the so-called liver-brain axis.^9-11^ This might link NAFLD to dementia, driven by the following mechanisms: (1) inflammation due to liver fat may activate microglial cells resulting in elevated expression of inflammatory cytokines in the brain^12^; (2) increased brain insulin resistance in patients with NAFLD may cause oxidative stress, excessive free fatty acids, and brain mitochondrial disorders^13^; and (3) cerebrovascular and hemodynamic disturbances provoked by a prothrombotic state.^8^ Despite this growing evidence for a liver-brain axis, current available studies reported no effects of NAFLD on dementia^14,15^ or only in frail participants with NAFLD with fibrosis.^16^ However, some other studies indicated that cognitive impairment was more common in patients with NAFLD^17^ or fibrosis,^18^ which might indicate a potential association with dementia and NAFLD.

The majority of those studies are, however, cross-sectional, had limited follow-up, or had a small sample size. Moreover, some studies lacked abdominal imaging to determine steatosis, and transient elastography was often not available to assess fibrosis. Given these limitations and the inconsistent results, the effect of NAFLD on dementia remains unclear. Therefore, we aim to study the associations of NAFLD and fibrosis with incident dementia and cognitive function in a well-defined, prospective cohort with available ultrasound and transient elastography data. A defining feature of our study is the use of different measures of NAFLD using various modalities that together provide a comprehensive assessment of liver function.

## Methods

### Participants

**Figure 1:**
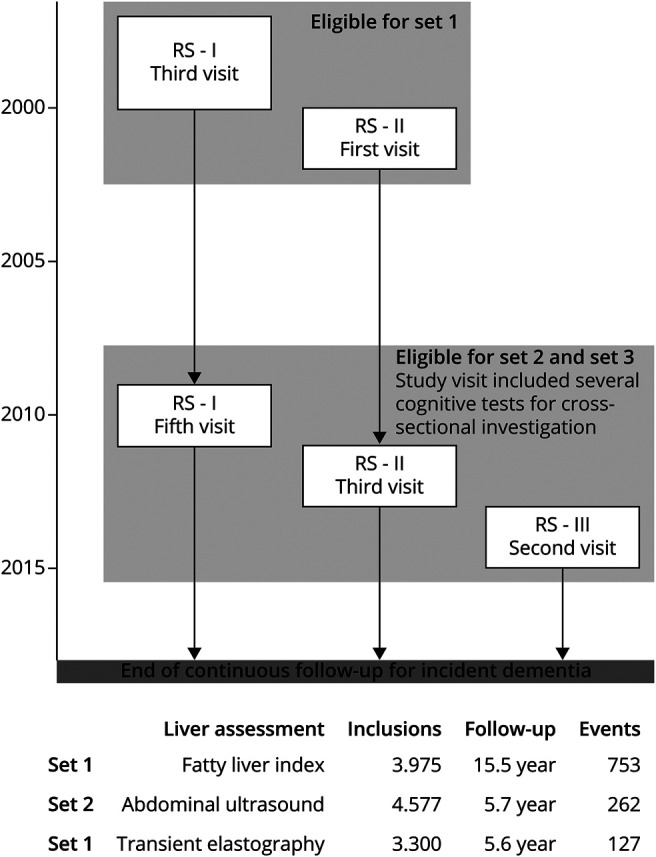
Overview of Different Study Sets and Key Characteristics for Investigating the Association Between NAFLD and Fibrosis With Dementia and Cognitive Function Set 1 and set 2 were used to study associations between NAFLD with incident dementia. Set 3 was used to study associations between liver stiffness and fibrosis with incident dementia. In addition, the effect of NAFLD and fibrosis on cognitive function was studied cross-sectionally in set 2 and set 3. NAFLD = nonalcoholic fatty liver disease.

This study was conducted within the Rotterdam Study, a prospective ongoing cohort that started in 1990. All individuals aged ≥45 years from a well-defined suburb in Rotterdam (Ommoord) were invited to participate in this longitudinal cohort designed to investigate chronic diseases in the general population. Several extensions to the cohort have been made over the years with an overall response rate of 72.0%.^19^ Study visits comprised a home interview and various physical examinations at the research center and were repeated every 4 to 6 years. In this study, we included 3 different sets (Figure 1) in which we assessed the effect of NAFLD or fibrosis on the risk of incident dementia in several ways. Set 1 comprised participants in whom we had available fatty liver index (FLI) to determine NAFLD, measured between 1997 and 2002. Set 2 comprised participants visiting the study center between 2009 and 2014 in whom we had abdominal ultrasound performed to assess NAFLD; this set comprised 40.3% of participants of set 1. Set 3 is a subset of set 2 and comprises participants who also underwent liver stiffness measurement (LSM) to assess fibrosis. Sets 2 and 3 were also used to investigate the association with cognition cross-sectionally.

Exclusion criteria were (1) prevalent dementia; (2) lack of follow-up; (3) missing dementia data; and (4) secondary causes for steatosis or missing alcohol data. These secondary causes were steatosis-inducing drug use, viral hepatitis, or excessive alcohol consumption (>20 g/d for female or >30 gr/d for male) assessed by food frequency questionnaire or alcohol interview.^20^ In addition, for set 3, participants with invalid LSMs were also excluded.

### Steatosis Assessment

NAFLD was defined as the presence of FLI ≥60 (set 1) or steatosis based on abdominal ultrasound (set 2) in the absence of secondary causes for steatosis. FLI was calculated with the following algorithm: FLI = (e^0.953^ × log_e_ (triglycerides) + 0.139 × BMI + 0.718 × log_e_ (GGT) + 0.053 × waist circumference − 15.745)/(1 + e^0.953^ × log_e_ (triglycerides) + 0.139 × BMI + 0.718 × log_e_ (GGT) + 0.053 × waist circumference − 15.745) × 100, where triglycerides were measured in mg/dL, GGT in U/L, waist circumference in cm, and BMI in kg/m^2^. Participants were categorized according to their FLI score as no NAFLD for FLI <30 and NAFLD for FLI ≥60.^21^ Steatosis based on abdominal ultrasound was defined as hyperechoic liver parenchyma compared with the spleen or kidney.^22^ Abdominal ultrasound was performed by a single certified and experienced sonographer on a Hitachi Hi Vision 900.

### Fibrosis Assessment

Liver stiffness was assessed using transient elastography (FibroScan, EchoSens, Paris, France). At least 10 measurements were obtained through either M or XL probe according to the device's instructions. Final measurements >7.1 kPa with an interquartile range >30% were considered unreliable and discarded.^23^ Liver fibrosis was defined as LSM ≥ 8.0 kPa.^24^

### Dementia Assessment

Dementia assessment was performed at baseline and subsequent center visits with the Mini-Mental State Examination and the Geriatric Mental Schedule.^25^ Those with a Mini-Mental State Examination score <26 or Geriatric Mental Schedule score >0 underwent further investigation including Cambridge Examination for Mental Disorders of the Elderly. Moreover, diagnosis of dementia by other health care professionals was available through electronic linkage of the study database with medical records from general practitioners and the regional institute for outpatient mental health care. An adjudication panel led by a consultant neurologist established the final diagnosis according to the standard criteria for dementia (*DSM-III-R*) for all sets and throughout the study period. Follow-up was complete until January 1, 2018. Within this period, participants were followed until the date of dementia, death, or January 1, 2018, whichever came first.

### Cognitive Testing

Besides the independent assessment of dementia, participants in set 2 and set 3 underwent several neuropsychological tests during the study visit; this includes the Stroop test, the Letter Digit Substitution Test (LDST), the Word Fluency Test (WFT), a 15-Word Learning Test with immediate and delayed recall, and Purdue Pegboard Test, which are described in eTable 1, links.lww.com/WNL/C90. These test results were transformed into a Z score, this reflects the number of SDs the test results were below or above the mean score. To assess the overall cognitive function, a general cognitive factor (G-factor) was calculated using principal component analysis. For this factor, we only included the LDST, WFT, WLTdel tests, and the trial 3 of Stroop test to prevent distortion of the G-factor by highly correlated tasks.^26^

### Covariates

Demographic and physiologic information was collected at baseline and included age, sex, education level (lower education, intermediate education, and higher education), smoking status (never, former, and current), alcohol intake (units/d), body mass index (BMI, kg/m^2^), alanine aminotransferase (U/L), and comorbidity (diabetes, hypertension, and stroke).^19^ Diabetes was defined as fasting glucose ≥7 mmol/L or use of antidiabetic drugs. Hypertension was defined as as systolic blood pressure ≥140 mm Hg, diastolic blood pressure ≥90 mm Hg or the use of antihypertensive medication. Presence of stroke was based on linkage with hospital records and verified by 2 experienced vascular neurologists. Depressive symptoms were assessed with a validated version of the Centre for Epidemiologic Studies Depression scale. Depression was defined as at least 16/60 points.^27^ *APOE* genotype was determined using a PCR and a biallelic TaqMan assay (rs7412 and rs429358) on labeled DNA samples. *APOE* ε4 allele represented carrier of 1 or 2 ε4 alleles.

### Statistical Analysis

Baseline characteristics are described for the overall population in all 3 sets. Data are expressed as mean ± SD or as median (with 25th–75th percentile [P25–P75]). For time-to-event analyses, we assessed the associations between NAFLD and liver stiffness with the risk of incident dementia using Cox proportional hazard regression analyses. Baseline was defined as the date of the blood test (for FLI) or abdominal ultrasound and follow-up ended at the diagnosis of dementia, death, or January 1, 2018. Model 1 was adjusted for *APOE* phenotype, age, sex, and education. Model 2 was in addition adjusted for alcohol, smoking, stroke, hypertension, diabetes, and cholesterol. Model 3 was in addition adjusted for BMI. Covariates above were selected based on previous literature, clinical relevance, and data availability.^28,29^ Missing genetic data were not imputed as they are innate and not modifiable; the remaining missing data were not imputed due to very low missingness (<2%).

Next, we determined the cross-sectional association of NAFLD or fibrosis with cognitive function using linear regression analyses and Tukey all-pair comparisons method based on analysis of variance models. We calculated the differences of the individual cognitive tests and G-factor for participants with NAFLD compared with those without NAFLD and for fibrosis compared with no fibrosis. Results were adjusted for age, sex, education level, smoking status, BMI, cholesterol, triglycerides, hypertension, stroke, diabetes, depression, and *APOE* genotypes.

A *p* value of <0.05 was considered statistically significant. All analyses were performed using R version 4.0.4 (Foundation for Statistical Computing, Vienna, Austria).

### Standard Protocol Approvals, Registrations, and Patient Consents

The Rotterdam Study has been approved by the Medical Ethics Committee of the Erasmus MC (registration number MEC 02.1015) and by the Dutch Ministry of Health, Welfare and Sport (Population Screening Act WBO, license number 1071272-159521-PG). The Rotterdam Study Personal Registration Data collection is filed with the Erasmus MC Data Protection Officer under registration number EMC1712001. The Rotterdam Study has been entered into the Netherlands National Trial Register (trialregister.nl) and into the WHO International Clinical Trials Registry Platform (who.int/ictrp/network/primary/en/) under shared catalog number NTR6831. All participants provided written informed consent to participate in the study and to have their information obtained from treating physicians. All authors had access to the study data and take full responsibility for the data, analyses, and interpretation of results.

### Data Availability

Data can be obtained on request. Requests should be directed toward the management team of the Rotterdam Study (secretariat.epi@erasmusmc.nl), which has a protocol for approving data requests. Because of restrictions based on privacy regulations and informed consent of the participants, data cannot be made freely available in a public repository.

## Results

### Baseline Characteristics

There were 3,975 participants with available NAFLD data based on FLI included in set 1, 4,577 participants with available ultrasound to assess NAFLD in set 2, and 3,300 participants with available LSM to assess fibrosis in set 3; exclusions are described in eTable 2, links.lww.com/WNL/C90. Participants from the different sets had a similar mean age (around 70 years) and BMI (near 27 kg/m^2^), and approximately 60% of them were women. In set 1, 1,293 (32.5%) participants had NAFLD (FLI ≥60), and in set 2, 1,586 (34.7%), which was based on abdominal ultrasound. In set 3, the median liver stiffness was 4.8 kPa (P25–P75: 3.8–5.9), and 192 (5.8%) participants had fibrosis (Table 1).

**Table 1:**
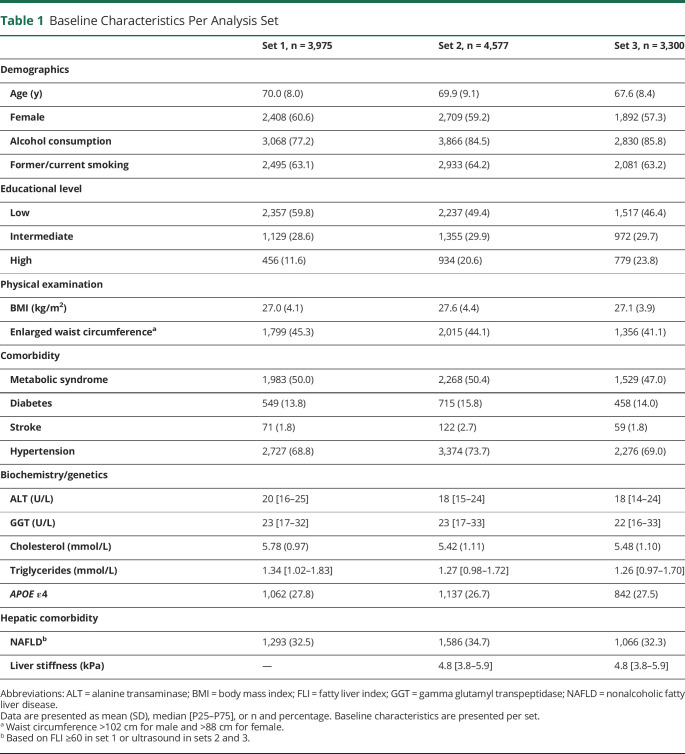
Baseline Characteristics Per Analysis Set

As shown in Figure 1, in set 1, 753 (18.9%) participants developed dementia during a median follow-up of 15.5 years. In set 2, the median follow-up was 5.7 years, and 262 (5.7%) participants had incident dementia. In set 3, only 127 (3.8%) had incident dementia with 5.6 years of median follow-up. Participants' characteristics stratified by NAFLD status for sets 1 and 2 are presented in eTable 3, and the characteristics stratified for fibrosis status (set 3) are available in eTable 4, links.lww.com/WNL/C90.

### NAFLD and Fibrosis in Relation to Incident Dementia

The presence of NAFLD (based on FLI ≥60, set 1) did not increase the risk of incident dementia (hazard rate [HR] 0.92; 95% CI 0.69–1.22) in the fully adjusted model. Similarly, no increased risk of dementia could be demonstrated for the presence of NAFLD, based on abdominal ultrasound in set 2. NAFLD was even associated with a significantly decreased risk for incident dementia in model 2 (HR 0.73, 95% CI 0.54–0.98), which was no longer significant after additional adjusting for BMI (HR 0.84; 95% CI 0.61–1.16). Consistent with those results, no association was found for fibrosis (HR 1.07; 95% CI 0.58–1.99) or liver stiffness (HR 1.01 per kPa; 95% CI 0.92–1.10) with incident dementia in fully adjusted models in set 3 (Table 2).

**Table 2:**
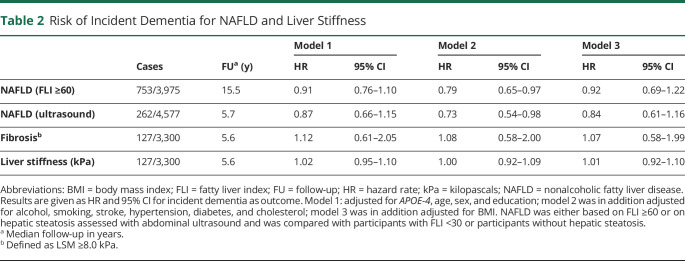
Risk of Incident Dementia for NAFLD and Liver Stiffness

Of interest, for the first 5 years of follow-up, participants with NAFLD (FLI ≥60, set 1) were at a significantly lower risk of incident dementia (HR 0.49; 95% CI 0.25–0.96) in the fully adjusted model, compared with no NAFLD (FLI <30). With the period of follow-up extending, the protective association between NAFLD and risk of incident dementia disappeared (between 5 and 10 years, HR 1.08; 95% CI 0.62–1.87; above 10 years, HR 1.25; 95% CI 0.80–1.96, Table 3).

**Table 3:**
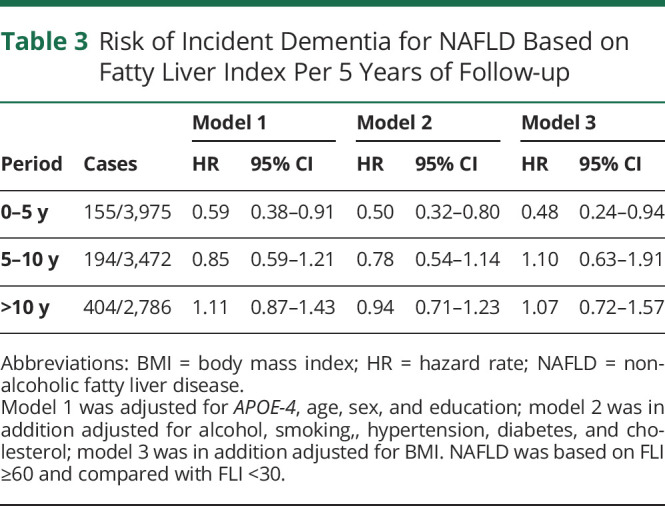
Risk of Incident Dementia for NAFLD Based on Fatty Liver Index Per 5 Years of Follow-up

Weight loss before abdominal ultrasound since the participants' previous visit (mean time between visits 6.1 years) was more evident among participants who had developed dementia during the follow-up, compared with those without incident dementia (mean: −0.37 vs −0.05 kg per year; set 2).

### NAFLD and Liver Fibrosis in Relation to Cognitive Performance

**Figure 2:**
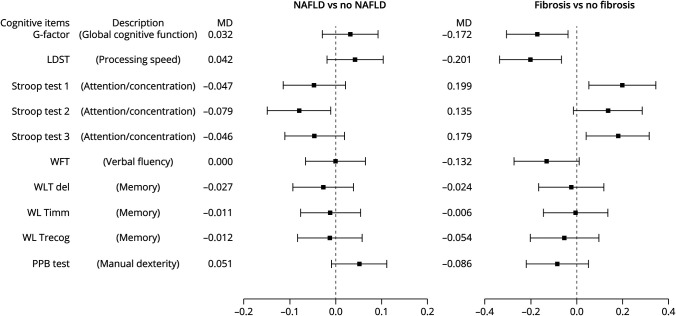
Mean Difference of Performance on Cognitive Tests Between Participants With NAFLD Compared With No NAFLD and Fibrosis Compared With No Fibrosis Expressed in Z-Scores Presence of NAFLD or fibrosis, in relation to cognition tests in cross-sectional analyses. Higher scores indicate better performance, except for the Stroop tests. Results were obtained from linear regression analyses and Tukey all-pair comparisons method based on ANOVA models. Differences were calculated for the individual cognitive tests and G-factor for participants with NAFLD compared with those without NAFLD and for fibrosis compared with no fibrosis. Results were adjusted for age, sex, education level, smoking status, BMI, cholesterol, triglycerides, hypertension, stroke, diabetes, depression, and *APOE* genotypes. G-factor = general cognitive factor; LDST = Letter Digit Substitution Test; MD = mean difference; PPB test = Purdue Pegboard Test; WFT = Word Fluency Test; WLTdel = Word Learning Test, delayed recall; WLTimm = Word Learning Test, immediate recall; WLTrecog = Word Learning Test, recognition.

Figure 2 presents the association of NAFLD (abdominal ultrasound, set 2) and liver fibrosis (set 3) with cognitive performance. Cross-sectional analyses revealed that NAFLD was not significantly associated with poor performance on global cognition reflected in G-factor (mean difference [MD] of Z score): 0.032 (95% CI −0.029 to 0.092); in fact, better performance of Stroop test 2 was observed in cross-sectional analyses. On the contrary, we found that liver fibrosis was associated with lower global cognition scores (MD −0.172, 95% CI −0.307 to −0.037) and lower scores of LDST and more time to finish Stroop tests 1 and 3 (eTable 5, links.lww.com/WNL/C90).

## Discussion

We investigated the effect of NAFLD on dementia and cognitive function in a large prospective ongoing population-based cohort with up to 15.5 years of median follow-up. NAFLD was not associated with an increased risk of incident dementia or impaired cognitive function. In addition, the presence of NAFLD was not associated with impaired cognitive function.

In contrast to the suggested liver-brain axis in previous studies, NAFLD did not increase the risk of incident dementia in this study, regardless of the modality of diagnosis (FLI or ultrasound). We even found NAFLD to be significantly protective for dementia within the first 5 years after FLI assessment. Similar trends were seen for the association between ultrasound-based NAFLD and incident dementia during the 5.7 years of median follow-up. This points us toward one of the challenges regarding NAFLD and dementia research: the reversibility of NAFLD due to weight loss.^30^ Dementia, albeit unintentionally, is also accompanied by weight loss during its preclinical phase,^31^ which was confirmed by our results. This could induce NAFLD regression, as even minor improvements in body fat have rather large effects on liver fat and hepatic triglycerides.^32,33^ Consequently, weight loss in the years before dementia could thus obscure any relation between NAFLD and incident dementia. In our study, the demonstrated protective effect of NAFLD on dementia disappeared after 5 years. This suggests that if NAFLD is associated with an increased risk for dementia at all, it is a long-term effect, and NAFLD itself might already have disappeared before dementia is diagnosed.

Given the reversibility of NAFLD, exposure duration could be of major importance to comprehend the association between NAFLD and dementia. Individuals with NAFLD can develop permanent liver fibrosis, resulting in higher liver stiffness, based on the duration and severity of NAFLD.^34^ Therefore, we assessed the association between fibrosis and liver stiffness with incident dementia longitudinally. In line with our results for NAFLD, fibrosis and liver stiffness were also not associated with incident dementia, indicating that neither NAFLD nor severity of NAFLD is associated with an increased risk for incident dementia. Considering cognitive impairment as a classic prodromal symptom preceding the onset of dementia.^33^ we explored the cross-sectional association between NAFLD and cognition independent of dementia. Similarly, we did not find a significant association between NAFLD and impaired cognitive function. However, fibrosis was significantly associated with impaired performance on the Stroop test and Letter Digit Substitution Test resulting in a lower G-factor score. These tests cover attention and concentration, processing speed, and global cognitive function, respectively. Further research is required whether this hints toward an association with dementia as well or is driven by common risk factors (e.g., the presence of diabetes or metabolic syndrome) or accumulation of toxins by impaired liver function.

Given these consistently negative results, we cannot demonstrate an association of NAFLD with dementia or cognitive function within our follow-up duration. This is in line with a recent registry study among over 40,000 participants, which could not link NAFLD and dementia using *ICD-10* codes.^14^ Moreover, a study with almost 20 years of follow-up could not identify NAFLD as risk factor for incident dementia.^15^ However, they reported that histology-proven fibrosis improved the prediction of dementia. Fibrosis was also linked to dementia among the frail elderly previously.^16^ However, these results need to be interpreted with caution because fibrosis was calculated based on age, which itself is undisputedly associated with dementia.

More literature is available on cognitive function, and in these studies, NAFLD has been linked to impaired performance on Serial Digit Learning Test^17^ and Symbol Digit Substitution Test,^17^ reduced reaction time,^17^ lower MoCA scores,^35,36^ brain volume reduction,^9^ and reduced brain activity.^36^ However, most results were unadjusted or disappeared after adjustment for important confounders such as age and education level. Moreover, most findings were not replicated, and some studies, similar to ours, could not identify any association with NAFLD and cognition.^18^ Therefore, the effect of NAFLD on cognitive function and dementia seems to be minor, if existing at all. In fact, in our study, we had 80% power to demonstrate an association between NAFLD and dementia for an HR of 1.25 in set 1 and an HR of 1.44 in set 2.

Although this study had a large sample size and extensive analysis was performed for both incident dementia and cognitive function in relation to NAFLD and fibrosis, the following limitations need mentioning. First, this cohort is almost entirely European, with a mean age of 70 years at baseline. Therefore, our results might not be generalizable to multiethnic and younger populations. Second, NAFLD and fibrosis were not based on liver biopsy because that procedure is invasive and subject to potential complications and therefore unethical to perform in a healthy population on this scale. Alternatively, we used FLI in set 1 and abdominal ultrasound in set 2. The FLI diagnosis correlates strongly with ultrasound diagnosis of NAFLD (AUROC 0.813) in the Rotterdam Study.^37^ Despite fully adjusted models, residual confounding might not be ruled out, as FLI includes BMI. In line with this limitation, NAFLD was only assessed at baseline, and no data were available for NAFLD exposure duration. Third, because we had only 192 cases of fibrosis, we might not have found an association with incident dementia. Therefore, the continuous outcome of liver stiffness was also used to explore associations with incident dementia; it should be noted, however, that this might not reflect only liver injury per se. Fourth, the cross-sectional study design for NAFLD and cognition allows not to study causal relationships for NAFLD on cognition. However, it served as indirect evidence for the absence of associations between NAFLD and dementia, in line with the longitudinal analysis. Finally, because NAFLD has clear associations with survival, survivor bias may have occurred. However, among the elderly, these effects are less obvious, and even protective effects of NAFLD on mortality have been observed; therefore, survivor bias is unlikely to have affected our results.^38-40^

In conclusion, individuals with NAFLD were not at an increased risk of dementia among this general elderly population, nor could an association with liver stiffness or fibrosis and dementia be demonstrated. Moreover, NAFLD was associated with a reduced risk of dementia for the first 5 years after the assessment, suggesting that NAFLD regression is likely before dementia onset, which could be driven by weight loss before dementia onset. As yet, NAFLD may have no clinical implications for dementia awareness. Further studies should focus on NAFLD exposure duration, NAFLD trajectory, and risk of dementia with longer follow-up durations.

## Glossary

BMI: body mass index
*DSM-III*: *Diagnostic and Statistical Manual of Mental Disorders, 3rd Edition*
FLI: fatty liver index
G-factor: general cognitive factor
HR: hazard rate
*ICD-10*: *International Classification of Diseases, 10th Revision*
LDST: Letter Digit Substitution Test
MD: mean difference
NAFLD: nonalcoholic fatty liver disease
PPB test: Purdue Pegboard Test
WFT: Word Fluency Test
